# Nanoscale measurements of unoccupied band dispersion in few-layer graphene

**DOI:** 10.1038/ncomms9926

**Published:** 2015-11-26

**Authors:** Johannes Jobst, Jaap Kautz, Daniël Geelen, Rudolf M. Tromp, Sense Jan van der Molen

**Affiliations:** 1 Huygens-Kamerlingh Onnes Laboratorium, Leiden Institute of Physics, Leiden University, PO Box 9504, Leiden NL-2300 RA, Netherlands; 2 IBM T.J. Watson Research Center, 1101 Kitchawan Road, PO Box 218, Yorktown Heights, New York 10598, USA

## Abstract

The properties of any material are fundamentally determined by its electronic band structure. Each band represents a series of allowed states inside a material, relating electron energy and momentum. The occupied bands, that is, the filled electron states below the Fermi level, can be routinely measured. However, it is remarkably difficult to characterize the empty part of the band structure experimentally. Here, we present direct measurements of unoccupied bands of monolayer, bilayer and trilayer graphene. To obtain these, we introduce a technique based on low-energy electron microscopy. It relies on the dependence of the electron reflectivity on incidence angle and energy and has a spatial resolution **∼**10** **nm. The method can be easily applied to other nanomaterials such as van der Waals structures that are available in small crystals only.

## Abstract (web summary)

The electronic properties of a material depend on both the filled and the unoccupied electron states. Here, the authors present a technique based on low-energy electron microscopy that is able to directly probe the unoccupied bands of few-layer graphene, as well as other materials.

## Graphical abstract

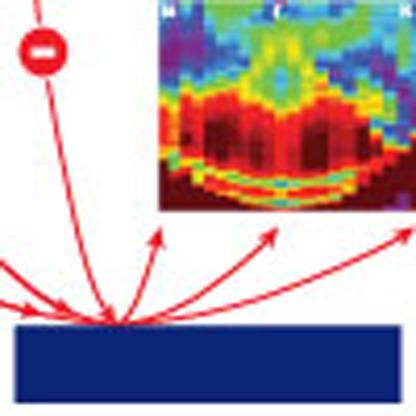

Due to their key importance in condensed matter physics, a variety of techniques has been developed to measure electron band structures^1^. Of these, angle-resolved photoemission spectroscopy (ARPES) is the most widely used method^2^. In ARPES, a sample is illuminated with photons (from lab ultraviolet-sources or synchrotron radiation) and the energy of the electrons that are released from the material due to the photoelectric effect^3^ is measured as a function of the in-plane electron momentum 
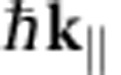
. It provides a high-energy resolution of up to 1 meV (ref. 4). In dedicated synchrotron-based ARPES facilities, a lateral resolution of up to 120 nm can be achieved by illuminating the sample with a strongly focused photon source^5^. Although ARPES has become a standard technique to measure occupied bands, probing unoccupied bands remains more challenging. One way to study them is k-resolved inverted photoemission spectroscopy (KRIPES). There, a surface is bombarded with low-energy electrons. As these electrons decay from one unoccupied band to another, photons are generated^6^. The energies of these photons are recorded and give information about the unoccupied bands^7^,^8^. This time-reversed version of the photoelectric effect suffers from very small cross-sections, causing the KRIPES intensity to be around five orders of magnitude smaller than ARPES signals^9^. The resulting long acquisition times make KRIPES rather painstaking and hence, not widely used. The high-electron beam currents required can also be problematic, potentially causing sample damage and/or contamination. Furthermore, very homogeneous samples are required as the probed area is typically larger than a square millimetre^10^. The problem of the low count rate in KRIPES is overcome in total current spectroscopy^11^,^12^ (TCS) and very-low-energy electron diffraction^13^ (VLEED) in which the absorption or reflectivity of low-energy electrons is directly measured, respectively. These methods have been used to determine the edges of unoccupied bands above the vacuum level in three-dimensional (3D) crystals^12^,^13^,^14^,^15^. Just like ARPES and KRIPES, however, they suffer from the fact that the probed area is large and local features are averaged out.

Here we present measurements of unoccupied bands above the vacuum level acquired using a technique that directly measures the in-plane dispersion relation of these bands from near-nanometre-size areas. Precise knowledge of these bands is an important part of material characterization and is essential for the detailed understanding of ARPES data^16^. Our technique follows the philosophy of VLEED: we use the energy-dependent reflectivity of low-energy electrons to measure unoccupied bands. In contrast to VLEED, where information is obtained from area-averaged diffraction patterns, we acquire the data from real-space low-energy electron microscopy (LEEM) images. Our method therefore is as elegant and robust as VLEED and, in addition, offers the high lateral resolution of LEEM (ref. 17), that is, its lateral resolution is five orders of magnitude better than existing techniques. Furthermore, it is, in terms of the probed energy range, complementary to KRIPES that primarily samples states below the vacuum level. High lateral resolution is crucial for studying novel nanomaterials that typically are either not available or not homogeneous on the millimetre scales needed for techniques such as TCS and VLEED. We demonstrate our new method by studying a sample with graphene domains (∼200 nm in diameter) of different layer number as a prototype van der Waals material^18^.

## Results

### Conventional low-energy electron microscopy

**Figure 1:**
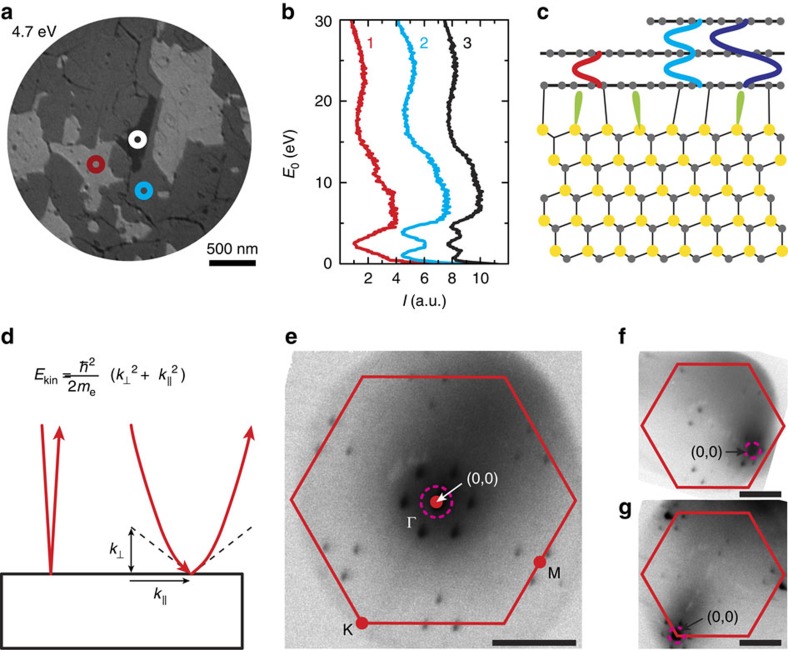
Principle of LEEM-based band structure measurements. (**a**) LEEM image (acquired at a landing energy of *E*_0_=4.7 eV) of monolayer (bright), bilayer (darker) and trilayer (darkest) graphene grown on SiC. (**b**) IV-curves, that is, reflected electron intensity as a function of landing energy *E*_0_=*E*−*E*_vac_, for monolayer (red), bilayer (blue) and trilayer (black) areas. The curves are shifted in intensity for clarity. The data are collected from single pixels indicated in **a**. (**c**) Schematic side view of SiC covered with monolayer and bilayer graphene (silicon atoms are shown in yellow, carbon atoms in grey). An electrically insulating buffer layer resides between SiC and the bottommost graphene layer. One interlayer state is formed for the monolayer graphene case between buffer layer and graphene (sketched schematically in red). For bilayer graphene, two of these interlayer states are found. They give rise to the minima in **b** (refs 24, 25). (**d**) Sketch of our experiment: In contrast to conventional LEEM (left), we introduce an in-plane momentum 
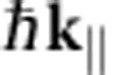
 of the electrons by tilting the electron beam (right). The kinetic electron energy (*E*_kin_) is related to 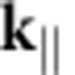 and the out-of-plane momentum 
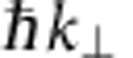
 via the vacuum dispersion relation. It determines the angle of incidence, which is equal to the angle of reflection, as well as the parabolic electron trajectories. (**e**) LEED analysis allows us to quantify 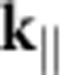. Here, the untilted case of 
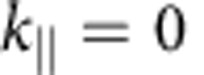
 is shown where the specular spot resides at the Γ-point in the center of the Brillouin zone (red hexagon). The dotted line indicates where the aperture is placed to detect only specularly reflected electrons (bright-field LEEM). (**f**,**g**) For the tilted cases, the central (0,0) spot is tilted towards the M-point and to the K-point, respectively. Scale bars in **e**–**g** correspond to 1 Å^−1^.

In a standard LEEM experiment, bright-field images (for example, Fig. 1a) are formed from the reflected intensity of a coherent beam of low-energy electrons, impinging normally onto the sample. The electrons are guided through the electron optics with an energy of 15 keV and are then decelerated towards the sample to energies of typically 0–50 eV. This is achieved by applying a decelerating voltage between objective lens and sample. By tuning this decelerating voltage, the landing energy *E*_0_ of the electrons, that is, the kinetic electron energy when interacting with the sample, can be selected precisely. For *E*_0_<0, the electrons are 100% reflected in front of the sample, that is, without touching it (mirror mode). Bright-field LEEM images (for example, Fig. 1a) are formed from the intensity *I* of specularly reflected electrons only^19^,^20^,^21^,^22^ by placing an aperture in the backfocal plane of the objective lens (indicated by dashed circles in Fig. 1e–g) to select the (0,0) diffracted beam. Thereby all electrons that are reflected under other angles (for example, Bragg reflections) as well as secondary electrons that complicate data interpretation are blocked. This particularly clean signal is a further advantage over other techniques.

A key feature of LEEM is that not only real-space images, but also local spectroscopic information can be obtained, because the low electron-landing energy matches the energy range of electronic states in typical materials^23^. In particular, electrons are absorbed if their landing energy *E*_0_ coincides with the energy of unoccupied states in the material. This increased absorbance manifests itself as a minimum in a so-called IV-curve, a measurement of the reflected intensity *I* versus *E*_0_, taken at a certain position^24^,^25^ (e.g. red curve in Fig. 1b).

### Low-energy electrons with non-zero in-plane momentum

In our technique, we deviate from standard LEEM by changing the tilt angle of the incoming electron beam (Fig. 1d), thereby introducing an in-plane momentum to the electrons. If both the energy *E*_0_ and the in-plane momentum 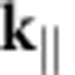 of the incoming electron match with an unoccupied band in the solid, the electron is absorbed with high probability and the reflectivity is low. In contrast, when there are no corresponding states in the solid (that is, the electron encounters a band gap), the reflectivity is high. Hence, by measuring IV-curves as a function of in-plane momentum, we determine the in-plane dispersion relation of unoccupied bands above the vacuum level. For 3D materials we measure, as for ARPES, the projection of the bands onto the 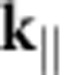-plane.

The degree of reflectance/absorbance of the electron beam depends not just on energy and 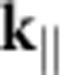, but also on the matrix element coupling the incoming and reflected vacuum electron plane waves with the specific electron band in the solid, its symmetry, density of states, and so on. Similar selection rules also apply to KRIPES, TCS and VLEED. Similarly, in ARPES there is an analogous transition of an electron from an occupied to an unoccupied state, which depends not just on photon energy, but also on the symmetry and polarization. The final transition in ARPES from the excited state to the vacuum plane wave state depends on the same coupling that underlies the present experiment. An advantage of the method presented here is that optical transitions play no role.

### Interlayer bands in van der Waals materials

As a starting point for our experiments, we show IV-curves measured for monolayer, bilayer and trilayer graphene, respectively (Fig. 1b). They are obtained from bright-field LEEM images (for example, Fig. 1a) at various energies with the electron beam normal to the surface. Focusing on the lower energies (<7 eV), we see one clear minimum for monolayer graphene, two for bilayer graphene and three for trilayer graphene. These minima are caused by the presence of unoccupied states between adjacent layers in these systems. For the material studied here, graphene on silicon carbide^26^,^27^,^28^,^29^ (SiC), the bottommost carbon layer is covalently bound to the SiC surface (Fig. 1c). Although this so-called buffer layer is electrically insulating, it does take part in the formation of interlayer states^25^. As a result, the number of interlayer states corresponds exactly to the number of conducting graphene layers. Hence, by analysing the minima in the IV-curves we can both determine the energy of interlayer states and the number of graphene layers^24^. We can use this layer- and energy-dependent reflectivity to create contrast-rich LEEM micrographs (Fig. 1a taken at *E*_0_=4.7 eV), where monolayer areas (one conducting graphene layer plus insulating buffer layer) appear bright while bilayer and trilayer graphene appear dark. Note that the IV-curves in Fig. 1b are measured locally (see markers in Fig. 1a), that is, each curve is taken from a single pixel, corresponding to a local area of 2.6 × 2.6 nm^2^. This is particularly exciting: we can measure the energy of unoccupied states with few-nanometre lateral resolution.

### Studying band dispersion by changing in-plane momentum

So far, we have performed LEEM in the conventional way (at normal incidence), investigating states with 
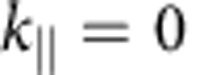
. This is equivalent to measuring the band structure at the Γ-point (centre of the Brillouin zone). To measure the full dispersion relation of the interlayer states, we want to determine their energy as a function of 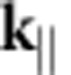. In bright-field LEEM the image is formed only from those electrons that are reflected specularly and elastically, that is, with conserved energy and in-plane momentum 
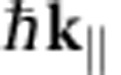
 under the condition of current continuity^16^ (upon reflection, the magnitude of the out-of-plane momentum 
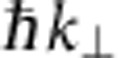
 is conserved while its sign reverses). This has the advantage that electrons with only a single angle of incidence and reflection are taken into account and hence, spherical aberrations do not deteriorate the resolution. This is further facilitated by the use of aberration-corrected electron optics (Supplementary Note 1). Therefore, one can study the interlayer states at different 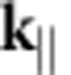 directly, by measuring IV-curves while changing 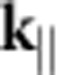 of the incident electrons. This is achieved using a beam deflector between electron gun and objective lens (Supplementary Fig. 1) that shifts the position of the incoming electron beam in the backfocal plane of the objective lens, thereby selecting the desired 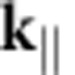. Owing to the decelerating field, which is perpendicular to the sample surface, the out-of-plane momentum 
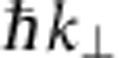
of the electrons is reduced on their way to the sample while the in-plane momentum 
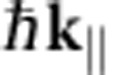
 is not affected. The electrons therefore, move on a parabolic trajectory determined by the vacuum dispersion 
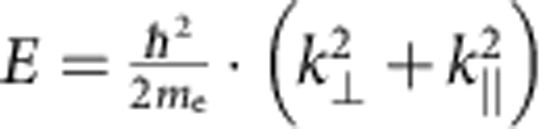
 with *m*_e_ the electron mass (see sketch in Fig. 1d). Consequently, for fixed 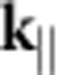, the incidence angle on the sample 
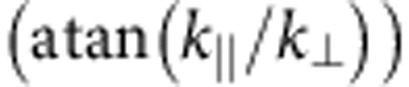
 is strongly dependent on the landing energy *E*_0_. Note that the sample is not moved, nor tilted, during this procedure and therefore, the same area is imaged for all tilt angles (Supplementary Note 2 and Supplementary Fig. 2). In fact, the resulting in-plane momentum 
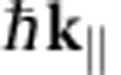
 can be accurately quantified from the angular distribution of reflected electrons, that is, the low-energy electron diffraction (LEED) pattern. Making use of LEED we are able to choose any value of 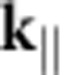 within the first Brillouin zone. To illustrate this, Fig. 1e shows the LEED pattern of the untilted (conventional) case, which corresponds to measurements at the Γ-point. Cases of large 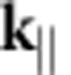, for measurements at the K-point and close to the M-point, are displayed in Fig. 1f,g, respectively.

### Band structure of few-layer graphene and graphite

**Figure 2:**
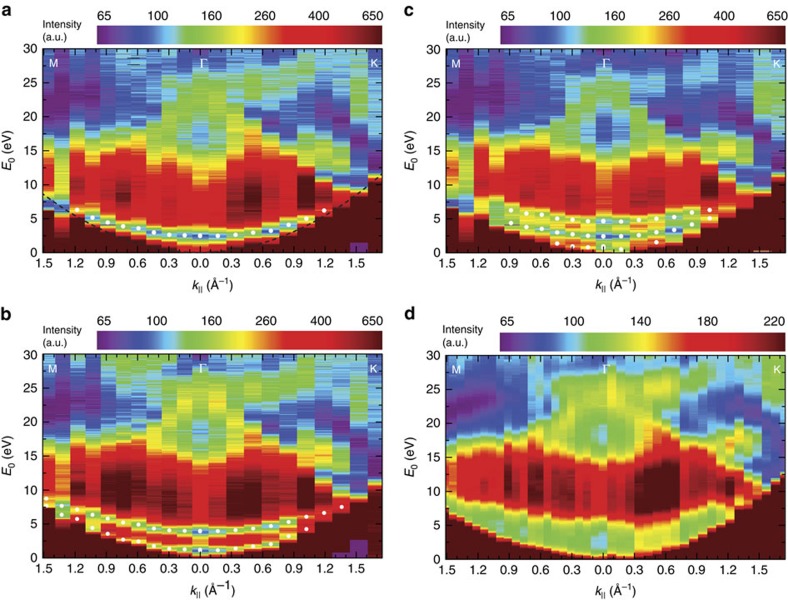
Electron reflectivity in a LEEM experiment as a function of in-plane momentum and energy. (**a**) Two-dimensional (2D) false-colour representation of IV-curves for different in-plane momenta 
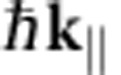
. The minimum in the IV-curves of monolayer graphene (narrow blue band near the bottom) shifts to higher energies for non-zero 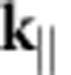. (**b**) Similar behaviour is observed for the minima (two blue bands) in bilayer graphene and (**c**) trilayer graphene. For all figures, 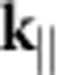 is varied from M to Γ to K. The data at the Γ-point are the IV-curves in Fig. 1b. White data points stem from Lorentzian fits to the individual IV-curves to determine the energetic position of the minima. (**d**) μVLEED measurement on an exfoliated graphite flake showing very similar global behaviour to **a**–**c**, apart from the discrete blue bands now being one continuous band. All four plots reflect the unoccupied 2D band structure of the respective material, where high reflected intensity (red) corresponds to band gaps and low intensity (blue) to electronic states in the solid that couple to the incoming/reflected plane wave electron beams. The dark red area at very low energies is formed by the mirror mode. Its curvature is described by the parabolic dispersion of electrons in vacuum (black dashed line in Fig. 2a).

To determine the 2D band structure along high-symmetry lines, we acquire LEEM images for landing energies between 0 and 30 eV, for 20 different 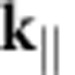-values in the M-Γ and the Γ-K-directions. For these 20 sets of images, IV-curves are measured from single-pixel areas (2.6 × 2.6 nm^2^). The images for different tilt angles are not perfectly aligned yielding a residual uncertainty of ∼10 nm, limiting the resolution of the technique at this time. Fig. 2a–c show colour plots of these 3D data sets for single pixels of monolayer, bilayer and trilayer graphene, respectively. Every column represents the IV-curve for a given 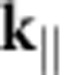. The data at the Γ-point (
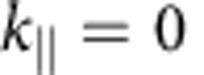
) are the IV-curves shown in Fig. 1b. The evolution of the minima in the IV-curves can be recognized in both maps as the narrow blue bands at low energies. As anticipated, one such band is visible for monolayer graphene (Fig. 2a), whereas bilayer graphene shows two (Fig. 2b) and trilayer three bands (Fig. 2c). All bands disperse upwards for non-zero 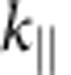, and eventually vanish when they touch the mirror mode boundary (dark red area in Fig. 2a–d). As argued above, the shape of these bands describes how the energy of the interlayer states of few-layer graphene depends on in-plane electron momentum. In other words, they are a direct measure of the band dispersion.

To benchmark the results in Fig. 2a–c, we also present a VLEED experiment, performed for a small graphite flake that was mechanically exfoliated^30^ and transferred onto a silicon substrate^31^ (Fig. 2d). For this, we have recorded LEED patterns while changing landing energy and in-plane momentum of the incident electrons. We use only the specularly reflected (0,0) spot for our analysis because it allows for the most direct interpretation. This is an advantage compared with conventional VLEED where the central spot is blocked by the electron gun^16^. Furthermore, we have been able to measure LEED images on an area (diameter ∼5 μm) much smaller than in conventional VLEED (ref. 13; ∼1 mm). Hence, this LEEM-based μVLEED method allows for investigations of micron-sized materials. Moreover, by inserting an illumination aperture, the diameter of the electron beam on the sample can trivially be reduced to 50 nm.

The unoccupied 2D band structures measured for monolayer, bilayer and trilayer graphene (Fig. 2a–c, respectively) show much similarity to the projected band structure of graphite (Fig. 2d). In particular, no additional substrate-related features are visible in Fig. 2a–c, showing that the interaction of the interlayer states with states in the SiC is negligible for the observed bands. In all four figures, the band gap between ∼7 eV and ∼15 eV (shown in red) is clearly resolved, while there are obvious differences at lower energies. Whereas in Fig. 2a–c, interlayer states lead to one, two or three well-defined resonance bands, respectively, we observe a continuous and broad band in the case of graphite (the lower green area in Fig. 2d), which is the projection of a 3D band onto the 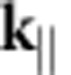-plane. The relation between this continuum and the discrete bands for few-layer graphene is discussed in the following.

## Discussion

**Figure 3:**
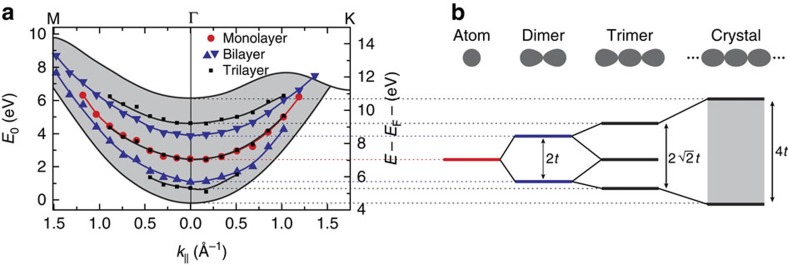
Unoccupied bands for monolayer, bilayer and trilayer graphene. (**a**) The IV-minima, shown as white circles in Fig. 2a–c, are plotted together with the calculated band, formed from interlayer states, for graphite (adapted from ref. 24). Red circles, blue triangles and black squares correspond to the data from the monolayer, bilayer and trilayer pixel indicated in Fig. 1a. The difference between the two energy axes with respect to the vacuum and Fermi level is given by the work functions of graphite^32^ Φ=4.6 eV. The observed behaviour can be understood in a tight-binding-like picture where the interlayer states resemble atomic orbitals. (**b**) Schematic that shows how an ‘atomic' monolayer state (red) splits into a ‘binding' and an ‘anti-binding' state (blue) and then in three trimer-states (black). The splitting energies of 2*t* and 
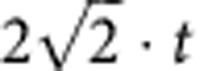
 are defined by the interaction strength *t* between different interlayer states. The continuous graphite band (grey area) of width 4*t* envelopes the quantized monolayer, bilayer and trilayer states. This is expected from the tight-binding picture, as graphite represents an infinite chain of interlayer states.

Figure 3a displays the measured values of the unoccupied interlayer bands (white circles in Fig. 2a–c) in monolayer, bilayer and trilayer graphene, together with a calculation of the band structure of graphite^24^. We show two energy axes, as the electron energy in band structures is typically referenced to the Fermi level *E*_F_, whereas the electron energy *E*_0_=*E*−*E*_vac_ in LEEM is defined with respect to the vacuum energy *E*_vac_. The difference between the two is the work function of graphite^32^ Φ=*E*_vac_−*E*_F_=4.6 eV (Supplementary Note 3 and Supplementary Fig. 3). Note that the data in Fig. 3a are measured directly from single pixels without any assumptions or calculations. Interestingly, the single monolayer band (red circles) lies between the two measured bilayer bands (blue triangles). In turn, they are all embedded in the calculated^24^, continuous band of graphite (shaded in grey). This behaviour can be surprisingly well understood in the framework of tight-binding theory, where a linear combination of local (for example, atomic) orbitals is used to calculate molecular orbitals and crystal band structures^33^,^34^. Here, the role of the local orbitals is played by the interlayer states. Single-layer graphene has one such state between the graphene plane and the buffer layer. For double layer graphene, however, two such interlayer states exist that couple in the *z*-direction, via a hopping integral *t*. The resulting hybridization yields two new eigenfunctions, that is, the even and odd combinations of the single interlayer states, with an energy difference of 2*t*. These states can be somewhat compared with the binding and anti-binding orbitals of a H_2_ molecule (cf. Fig. 3b), with the difference that the graphene states are planar, having a continuous dispersion in the in-plane directions. For trilayer graphene, a splitting of 
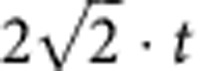
 is expected. In our experiment, we find splittings of 2.8 eV and 3.9 eV for bilayer and trilayer, respectively. This consistently yields *t*=1.4 eV in agreement with calculations in literature^25^. Note that the low-energy minima for trilayer graphene lie close to the mirror mode transition (cf. Fig. 2c). Hence, they are more difficult to determine and their energy might be slightly overestimated. Finally, bulk graphite contains a macroscopic number of interlayers that couple to form a continuous band, as indeed observed as the green area at lower energies in Fig. 2d. The expected band width in tight binding is 4*t*, that is, twice the double layer splitting. This is consistent with Fig. 3a, making the figure an elegant example of tight-binding theory.

In summary, we measure unoccupied bands of monolayer, bilayer and trilayer graphene on SiC by introducing a novel LEEM-based technique. In a nut shell, we record the specularly reflected electron intensity as a function of tilt angle (that is, in-plane momentum) and energy of the incoming electrons. Data are acquired from real-space LEEM images and therefore allow for near-nanometre lateral resolution. This enables us to study unoccupied bands on inhomogeneous samples. Moreover, these bands, to which the incident electron plane waves couple, are measured directly, without any need for complex data analysis or additional assumptions. The measured bands of few-layer graphene are compared with calculations and provide an elegant visualization of the tight-binding concept. The technique is in principle straightforwardly applicable to any material, most prominently the ever growing family of quasi-2D van der Waals materials with its pronounced resonance-mediated features in the LEEM IV-curve. Specifically, it is perfectly suited for small flakes, as typically obtained from mechanical exfoliation techniques.

In the future, this novel technique will allow one to additionally investigate quasiparticle lifetimes and related many-body effects, from the intensity and width of the observed features, as well as higher-order resonances in free-standing graphene^35^. Now, measurements of unoccupied band structures on nanometre length scales can be performed in every LEEM/photoemission electron microscopy (PEEM) system, complementing occupied band measurements using ARPES. We note, however, that spatial resolution is better preserved in aberration-corrected LEEM systems as used here. Thus, for the first time, we can analyse both occupied and unoccupied states, with high spatial and momentum resolution, on a single sample, in a single instrument. Moreover, recent developments in spin-polarized electron sources for LEEM (refs 22, 36) can directly be used to study the spin-polarized band structure in the near future, using the method presented here.

## Additional information

**How to cite this article:** Jobst, J. *et al.* Nanoscale measurements of unoccupied band dispersion in few-layer graphene. *Nat. Commun.* 6:8926 doi: 10.1038/ncomms9926 (2015).

## Supplementary Material

Supplementary Information Supplementary Figures 1-3, Supplementary Notes 1-3 and Supplementary References
