# Supplementary material for: Nanoscale measurements of unoccupied band dispersion in few-layer graphene
Source: Nat Commun. 2015 Nov 26;6:8926. doi: 10.1038/ncomms9926 (PMC4674768; doi:10.1038/ncomms9926)
Supplement: Supplementary Information — Supplementary Figures 1-3, Supplementary Notes 1-3 and Supplementary References [file ncomms9926-s1.pdf]

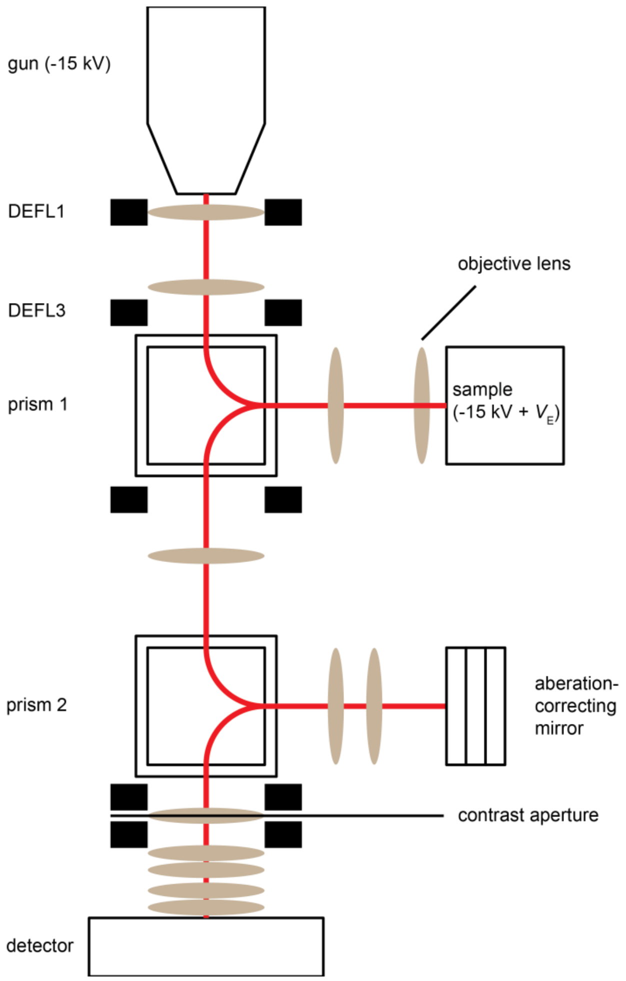

Supplementary Figure 1| Schematic drawing of the used LEEM setup. Electrons (red line) travel from an electron gun via a magnetic prism towards the sample. They are decelerated between objective lens and sample, where they reflect. Via an aberration-correcting mirror they are projected onto a detector. All lenses are shown in gray and electrostatic deflectors as black rectangles. We use the deflector DEFL1 to tilt the electron beam in order to introduce an in-plane momentum for our experiments. DEFL3 is used to bring the electron beam back to the center of the field of view. The contrast aperture, used for bright-field LEEM is indicated.

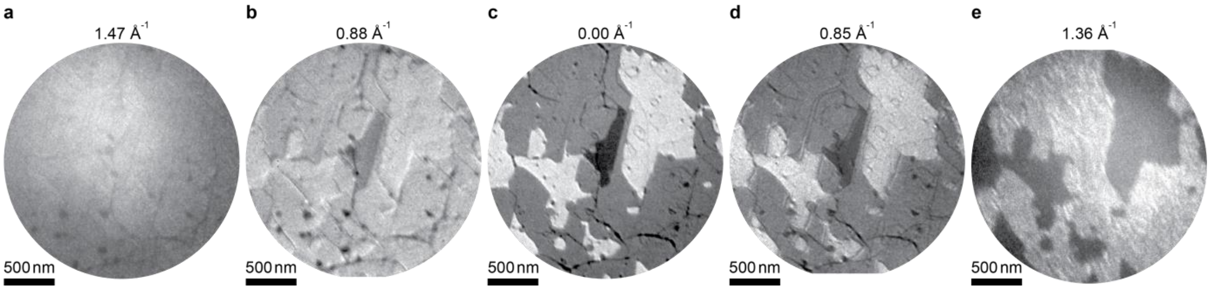

Supplementary Figure 2| LEEM images for different values of $k_{||}$. The LEEM images show the same area of the sample for the full range of $k_{||}$. The applied in-plane momentum ranges from **a,** the M-point with $k_{||} = 1.47$ Å^{-1}, via **b,** $k_{||} = 0.88$ Å^{-1}, **c,** the Γ-point with $k_{||} = 0$ and **d,** $k_{||} = 0.85$ Å^{-1} to **e,** 80% to the K-point with $k_{||} = 1.36$ Å^{-1}. The landing energies E_0 are 27.7 eV, 9.1 eV, 6.5 eV, 8.0 eV and 12.0 eV, respectively. The different contrast arises due to the different IV-curves. For large in-plane momentum ($k_{||} > 1.4$ Å^{-1}) contrast-rich images can no longer be accomplished.

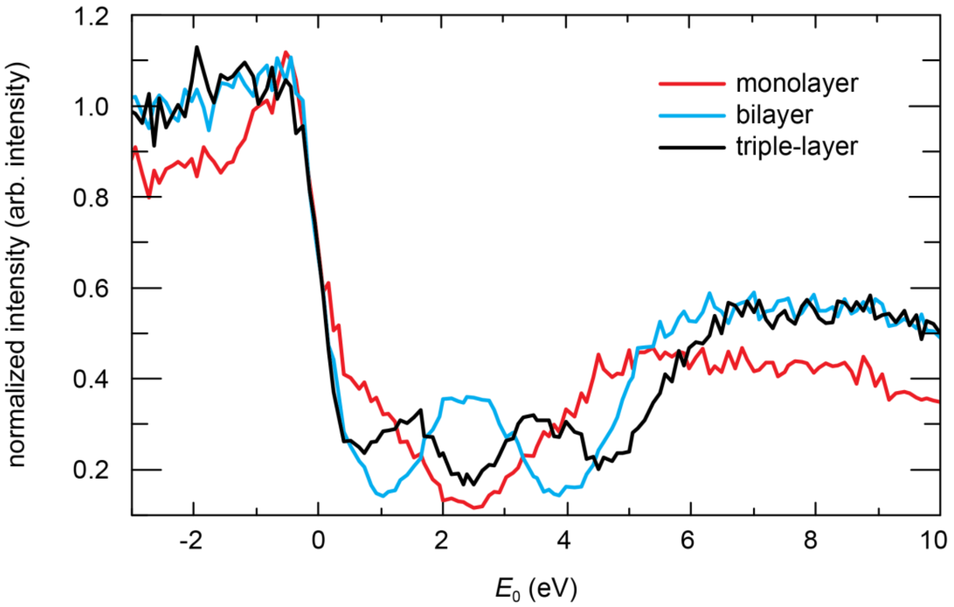

Supplementary Figure 3| IV-curves for different layer thickness. The mirror-mode transition (MMT) is clearly visible as steep drop at $E_0 = 0$ in IV-curves, occurring at the same energy for monolayer, bilayer and trilayer graphene. This indicates that the three materials have the same work function Φ. The plotted curves (obtained from single pixels in the images) are the same as in Fig. 1b in the main manuscript but are normalized to the mirror mode intensity and are not shifted in intensity.

Supplementary Note 1. Low-energy Electron Microscopy (LEEM)

We use the ESCHER setup, an aberration-corrected SPECS FE-LEEM P90 instrument, for the investigations described. In this instrument (see sketch in Supplementary Figure 1 and detailed description in Ref. [1] and [2]), electrons are produced in an electron gun (with cold-field emitter) and – accelerated to 15 keV – they propagate through the imaging system of the microscope. They are deflected towards the sample via a magnetic prism array (prism1 in Supplementary Figure 1). Between objective lens and sample, the electrons are decelerated to low energies (typically 0 to 50 eV) by an electric field. After reflection off the sample, they are accelerated back

into the lower part of the imaging system through the objective lens by the same field. After passing the first prism array, they are deflected onto a mirror with a second prism array (prism2 in Supplementary Figure 1). This mirror consists of three plates at different electrical potential and corrects for spherical and chromatic aberrations introduced by objective lens. It allows us to reach a resolution of 1.4 nm.[3] The electrons are then projected onto a detector that consists of a channel plate and a CCD camera.

For bright-field LEEM investigations, a contrast aperture is inserted into a back focal plane to select only specularly reflected electrons (the central spot in the LEED pattern of the sample).

In order to tilt the electron beam and thereby introduce an in-plane momentum $k_{||}$ to the electrons, we use an electrostatic deflector (DEFL1 in Supplementary Figure 1). Note that in conventional LEEM, DEFL1 is actually used to align the system for perpendicular electron incidence ($k_{||} = 0$). We use a second electrostatic deflector, DEFL3, to keep the tilted electron beam in the center of the field of view.

Supplementary Note 2. Effect of In-plane Momentum on Imaging Conditions

By using an aberration-corrected microscope we can keep the same area of the sample in the field of view for the whole course of the experiment. Still a slight drift of the image occurs while changing the landing energy. This is subsequently compensated by a drift-correction script. Supplementary Figure 2 shows the studied area for five different tilt angles/in-plane momenta. It is apparent that we can, indeed, choose a spot on the sample and investigate it for all tilt angles. The contrast within the images is varies

from image to image because the IV-curves change with changing in-plane momentum (cf. Fig. 2 in the main manuscript).

Using non-corrected systems, the illuminated area on the sample can always be kept constant, but image-shift corrections are necessarily significantly larger. We also note that spatial resolution is better preserved in aberration-corrected LEEM systems.

Supplementary Note 3. Work Function Measurement for Monolayer, Bilayer and Trilayer Graphene

Due to the low electron energies used, LEEM is particularly sensitive to surface charge. Consequently, it is routinely used to measure work function differences in materials. This is done by measuring the energy at which the mirror-mode transition (MMT) occurs, i.e. the landing energy at which electrons start to interact with the sample. This energy can easily be identified as the point where the electron intensity in the IV-curve drops abruptly. This energy is defined as $E_0 = 0$ and electrons with $E_0 < 0$ do not reach the sample surface. If materials have different work functions, the electrons are decelerated differently and thus, the MMT in the IV-curve shifts.

Supplementary Figure 3 shows IV-curves obtained from monolayer, bilayer and trilayer graphene pixels. The MMT for the three materials is clearly identical. Hence, we conclude that monolayer, bilayer and trilayer graphene in our experiment have identical work functions in contrast to earlier reports on similar materials[4,5]. Figure 3a in the main manuscript also indicates identical work function for different graphene layer thickness.

Supplementary References

1. Schramm, S. M. *et al.* Low-energy electron microscopy and spectroscopy with ESCHER: Status and prospects. *IBM J. Res. Dev.* **55,** 1:1–1:7 (2011).

2. Tromp, R. M., Hannon, J. B., Wan, W., Berghaus, A. & Schaff, O. A new aberration-corrected, energy-filtered LEEM/PEEM instrument II. Operation and results. *Ultramicroscopy* **127,** 25–39 (2013).

3. Schramm, S. M. PhD dissertation (Leiden University): Imaging with Aberration-Corrected Low Energy Electron Microscopy. (2013).

4. Hibino, H. *et al.* Dependence of electronic properties of epitaxial few-layer graphene on the number of layers investigated by photoelectron emission microscopy. *Phys. Rev. B* **79,** 125437 (2009).

5. Filleter, T., Emtsev, K. V., Seyller, T. & Bennewitz, R. Local work function measurements of epitaxial graphene. *Appl. Phys. Lett.* **93,** 24–26 (2008).
